# AGING IN THE RIGHT PLACE: HOUSING AND SOCIAL SUPPORTS FOR OLDER ADULTS EXPERIENCING HOMELESSNESS

**DOI:** 10.1093/geroni/igad104.0205

**Published:** 2023-12-21

**Authors:** Anthony Traver, Holly Dabelko-Schoeny

## Abstract

The Aging in the Right Place (AIRP) framework recognizes that secure housing for older adults should support one’s unique vulnerabilities and lifestyles. Applying the AIRP framework to older people experiencing homelessness (OPEH) reveals limited access to the types of living environments and supportive services that promote dignity, health, and inclusion in later life. This symposium aims to use the AIRP framework to examine the places, meanings, and opportunities experienced by diverse groups of OPEH striving to secure housing and promote their well-being. The first paper uses photovoice interviews and thematic analysis to broaden the current conceptualization of AIRP by exploring how feelings, actions, and place inform the meaning of AIRP for older adult residents of one temporary housing program in Vancouver, CA. The second examines the housing and support needs of older adult veterans in Calgary, CA residing in temporary congregate housing through a multi-methods approach involving document reviews, environmental audits, and in-depth interviews. The third paper uses interviews with OPEH and service providers in Columbus, OH to look across the continuum of housing occupied by older adults before, during, and after an episode of late-life homelessness and to investigate the mechanisms that allow OPEH to identify and access the housing they consider optimal. Finally, the discussion positions AIRP for OPEH within the broad context of Age-Friendly ecosystems to highlight the role of coordination between housing providers, government leaders, health services, university researchers, and residents to ensure that AIRP is achievable for precariously housed older persons.

